# *In Vivo* Flow Cytometry of Circulating Tumor-Associated Exosomes

**DOI:** 10.1155/2016/1628057

**Published:** 2016-11-14

**Authors:** Jacqueline Nolan, Mustafa Sarimollaoglu, Dmitry A. Nedosekin, Azemat Jamshidi-Parsian, Ekaterina I. Galanzha, Rajshekhar A. Kore, Robert J. Griffin, Vladimir P. Zharov

**Affiliations:** ^1^Department of Otolaryngology-Head and Neck Surgery, University of Arkansas for Medical Sciences, Little Rock, AR 72205, USA; ^2^Arkansas Nanomedicine Center, University of Arkansas for Medical Sciences, Little Rock, AR 72205, USA; ^3^Department of Radiation Oncology, University of Arkansas for Medical Sciences, Little Rock, AR 72205, USA

## Abstract

Circulating tumor cells (CTCs) demonstrated the potential as prognostic markers of metastatic development. However, the incurable metastasis can already be developed at the time of initial diagnosis with the existing CTC assays. Alternatively, tumor-associated particles (CTPs) including exosomes can be a more valuable prognostic marker because they can be released from the primary tumor long before CTCs and in larger amount. However, little progress has been made in high sensitivity detection of CTPs, especially* in vivo*. We show here that* in vivo* integrated photoacoustic (PA) and fluorescence flow cytometry (PAFFC) platform can provide the detection of melanoma and breast-cancer-associated single CTPs with endogenously expressed melanin and genetically engineered proteins or exogenous dyes as PA and fluorescent contrast agents. The two-beam, time-of-light PAFFC can measure the sizes of CTCs and CTPs and identify bulk and rolling CTCs and CTC clusters, with no influence on blood flow instability. This technique revealed a higher concentration of CTPs than CTCs at an early cancer stage. Because a single tumor cell can release many CTPs and* in vivo* PAFFC can examine the whole blood volume, PAFFC diagnostic platform has the potential to dramatically improve (up to 10^5^-fold) the sensitivity of cancer diagnosis.

## 1. Introduction

Most deaths from cancer (up to 90%) result from metastases for which there are no effective therapies [1–5]. Studies performed in our and other laboratories have demonstrated the tremendous potential of circulating tumor cells (CTCs) as a prognostic marker of metastatic development and therapeutic efficacy [6–14]. Currently available advanced CTC assays (e.g., CellSearch and microfluidic CTC chips, among others) [10] have provided many biological discoveries that include high CTC heterogeneity, the presence of tumor-initiating and dormant cells, CTC epithelial-mesenchymal transition, and CTC-emboli with high metastatic activity. However, despite the enormous efforts in the development of new CTC assays, the principle limitation of all existing technologies is the inherently low sensitivity of detection at around 1–10 CTCs/mL, which is primarily due to the sampling of a small blood volume (1–10 mL). As a result, the existing CTC assays can miss up to 10^3^–10^4^ CTCs (i.e., 99.9% of CTCs) in the entire blood volume, one of which could easily drive metastatic progression to an incurable stage before CTCs can be detected with existing assays [10]. Because existing CTC assays cannot provide early enough cancer diagnosis, it is possible that it is too late to treat a patient at the time of initial testing.

In addition to CTCs, tumors that secrete extracellular vesicles, which include exosomes, nanoparticles, and microparticles which will collectively be referred to here as circulating tumor-associated particles (CTPs), can harbor tumor cell signatures associated with local and metastatic progression (e.g., membrane proteins and microRNA) [15–32]. In particular, exosomes with typical sizes of 30–300 nm have endosomal origins [15], whereas microparticles, including microvesicles 100 nm–1 *µ*m in size, are released directly from the plasma membrane [33]. Because CTPs can be released from the primary tumor long before CTCs and are vastly more abundant, CTPs have the potential to be a valuable prognostic tool. A single tumor cell may produce thousands of CTPs. Thus, unlike CTCs, CTPs can be present in plasma at very high concentrations (e.g., in some cases up to 10^10–12^ CTPs/mL versus 1–10^2^ CTCs/mL) and could also be used as markers of nonmetastatic cancers when no CTCs are present in blood. CTPs can also traffic between cellular populations to help create important premetastatic niches [19].

However, little progress has been made in assessing the diagnostic value of CTPs because of the many challenges associated with their small sizes (30 nm–1 *µ*m), biological diversity (i.e., origins, structures, functions, biogenesis, and secretion mechanisms), and the limitation of detection methods [23–32]. For example, among different detection methods (e.g., RT-PCR, cytometry, Raman microspectroscopy, and transmission electron microscopy (TEM)), Western blot and chemiluminescence ELISA have demonstrated the best detection limits of 10^5^–10^7^ CTPs (exosomes) in a sample of only a few mL. A new nanoplasmonic sensor improved this limit to 3000 CTPs [29], which is still ~10^3^-fold lower than the threshold for CTCs (~1 CTC/mL). In brief, there are no methods that currently exist for the selective detection and identification of CTPs in circulation* in vivo*. However, the recent advances in the development of a multimode photoacoustic (PA) and fluorescence flow cytometry (PAFFC) [34–41] provided a unique diagnostic platform to detect and identify CTPs directly in the bloodstream. This platform opens up the opportunity for the examination of nearly the entire volume of blood (up to 3–5 L). This allows for a dramatic increase in sample volume and hence chances to detect rare circulating disease-associated markers, compared to conventional blood tests involving blood samples of just a few mL. A combination of CTC and CTP detection may dramatically (100–1000-fold) increase the sensitivity of* in vivo* flow cytometry due to a tumor's potential ability to release up to 1000-fold more CTPs than CTCs. Here, we demonstrate the first proof of this concept using the* in vivo* flow cytometry platform for detection of CTPs at early disease stages.

## 2. Materials and Methods

### 2.1. Photoacoustic (PA) Flow Cytometry and Fluorescence Flow Cytometry (PAFC and FFC, Resp.) Platform

**Figure 1:**
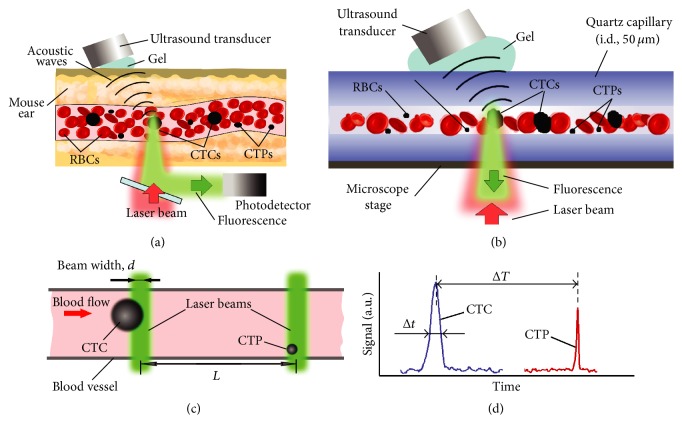
Photoacoustic and fluorescent flow cytometry platform. (a) The principle of* in vivo* detection of CTCs and CTPs using integrated PAFFC schematic. (b) The* in vitro* schematic for detection of CTCs and CTPs in a capillary tube using PAFC. (c) Two-beam time-of-flight schematic. (d) Signal diagram for CTC and CTP identification in two-beam time-of-flight mode.

 Principles of PAFC and FFC as well as their integration (PAFFC) were described in detail elsewhere [34–42]. Briefly, one or several laser beams irradiate a circulating object directly in blood flow. This produces PA waves or fluorescence light (referred to as PA and fluorescence signals) that are detected with an ultrasound transducer and photodetector, respectively (Figure 1(a)). Skin and many red blood cells (RBC) in the detection volume create constant background signals. To be detectable, individual targets (i.e., CTCs and CTPs) must have higher localized absorption and fluorescence than background signals. The integrated PAFFC setup was based on Nikon Eclipse E400 microscope platform (Nikon Instruments Inc., USA) with a high pulse repetition rate (10 kHz) nanosecond (0.6–8 ns) lasers operating at 532 nm and 820 nm (LUCE 532, LUCE 820, and Bright Solutions, resp.) for PA detection of CTPs with endogenously expressed melanin or labeled with nanoparticles as PA contrast agents and a continuous wave 488 nm laser diode (Power Technologies, Alexander, AR) for fluorescence detection of CTPs expressing green fluorescence protein (GFP) or labeled with fluorescence tags. Laser beams were focused on sample into a 6 *µ*m × 60 *µ*m line using a cylindrical lens and 40x objective (Plan Fluor, NA 0.65; Nikon Instruments). The same objective collected fluorescence from GFP or fluorescence labels. PA signals from circulating objects were detected by an ultrasound transducer (model 6528101, 3.5 MHz, 5.5 mm in diameter; Imasonic Inc., Besancon, France) and then amplified (amplifier model 5662B, 50 kHz–4 MHz; Panametrics). To collect signals, the setup was equipped with an analog-to-digital converter board (PCI-5124, National Instruments, Austin, TX) controlled using custom* LabVIEW* software. PA signals were sampled at 200 M samples/s with 12-bit resolution. Fluorescence signals from a photomultiplier tube were sampled at a rate of 4 MHz and downsampled to 10 kHz with 400 points averaging. All the data was presented as signal traces in which amplitudes, shapes, and widths for each transient peak exceeding the background level were analyzed with customized software.

### 2.2. *In Vitro* PAFFC Analysis of CTCs and CTPs

*In vitro* PAFFC was used to verify* in vivo* data because there is a higher sensitivity* in vitro* due to low light attenuation, autofluorescence, absorption background, well-controlled flow parameters, and sample dilution to exclude overlapping peaks from closely located objects. Specifically,* in vitro* analysis of CTPs and CTCs was performed in 50 and 100 *µ*m (i.d.) square quartz capillary tubes in phosphate-buffered saline (PBS) or blood. The flow velocity of 1–5 mm/s was controlled with a syringe pump (Figure 1(b)). To ensure that only one cell/particle is in the detection volume at a time, the solution of cells or particles were diluted properly. PA and fluorescence signals were detected through the capillary walls using the integrated PAFFC system as described above. PA and fluorescence traces were analyzed to identify a transient increase in PA or fluorescence signal amplitudes during a short time window (typically ~0.5 ms). The 2D scatter plots are constructed as PA signal amplitude versus fluorescence intensity with each object presented as a single dot.

### 2.3. *In Vivo* Flow Cytometry Using Two-Beam Time-of-Flight Schematics

The principle of the time-of-flight flow cytometry technique using preferentially one laser beam was reported previously [38–40]. Briefly, in a modified schematic, a CTP or CTC traverses two linear laser beams in the vessel (Figure 1(c)) separated by a fixed distance (20–40 *µ*m). During passing each laser beam the objects in flow produce a signal detected by the corresponding detector (Figure 1(d)). Thus, the same object appears with a certain time shift on two data traces. Since the distance between the excitation laser beams is well established, the time shift in the data traces gives the exact cell velocity in flow. Secondly, the width of the cell signal (the time it takes for a cell to travel through a single laser beam) depends on the laser beam width, object velocity, and size. Thus, only an object's size is unknown, as the laser beam width can be calibrated using objects of a known size (e.g., beads). The combination of these two approaches provides a full data set for each circulating object and this allows us to identify its size and velocity in flow. This mode can be applied to cells with endogenously expressed contrast agents (e.g., with melanin) or cells targeted by exogenous labels (e.g., nanoparticles). As CTPs have different structural and molecular compositions depending on the cancer type and stage, the analysis of width and shape of PA and fluorescence signals provide specific CTP's size which is distinctive from CTC parameters.

### 2.4. Cells

Mouse melanoma (B16F10), human breast cancer (MDA-MB-231), and rat adenocarcinoma (MTLn3) cell lines expressing GFP and Dendra2 were employed to provide opportunities for the monitoring of CTCs and CTPs* in vitro* in PBS and in the xenograft of a nude mouse model (below). The cell lines were purchased from ATCC and were cultured in Dulbecco's Modified Eagle Medium (DMEM) with high glucose (Gibco) + 10% Fetal Bovine Serum (FBS) and penicillin-streptomycin. The MTLn3 adenocarcinoma cells expressing Dendra2 protein were maintained in a minimum essential medium (Invitrogen/Life Technologies) with 10% FBS and penicillin-streptomycin (Invitrogen/Life Technologies). Viable cells were resuspended in PBS to the desired concentration.

### 2.5. Nanoparticles

Gold nanorods (GNRs) were from Nanopartz (Loveland, CO) with an absorption maximum at 820 nm (GNR-820). These nanoparticles follow the main requirements for targeting of CTCs and CTPs: small sizes (≤40–60 nm), low toxicity with polyethylene glycol-coating (PEG), high near infrared (NIR) absorption, and easy conjugation. Specifically, GNR-820 were conjugated with folic acid to target surface folate receptors expressed in breast cancer cells and not normal blood cells.

### 2.6. Animals

Animals were used in accordance with a protocol approved by the University of Arkansas for Medical Sciences Institutional Animal Care and Use Committee. Nude mice (nu/nu), 8–10-week-old, weighing 20–30 g, were procured from a commercial source for use in the experiments. The animals were anesthetized by isoflurane and placed on a heated microscope stage at 38°C (body temperature). To create a primary tumor with metastatic dissemination, mice (*n* = 8) were inoculated with 1–3 × 10^6^ Dendra2-MTLn3 cells in an ear. The mice were examined every other day with PAFFC to detect the appearance of CTPs and CTCs.

Subcutaneous inoculation of 1 × 10^6^ tumor cells (B16F10 and MDA-MB-231) provided the tumor model on the mouse's back. For* in vivo* monitoring of CTCs and CTPs, mice were anesthetized using isoflurane inhalation (1.2%). The mouse ear was spread over the stage glass window and measurements were performed in 40–60 *µ*m ear vessels. All experimental sessions consisted of continuous monitoring of CTCs and CTPs using the PAFFC system. The transducer was placed gently on the skin close to the laser beam with transparent ultrasound gel used for acoustic coupling. Mouse blood was collected from the tail vessel for* in vitro* testing using* in vitro* PAFFC and fluorescence microscopy. A total of five animals were used for each experiment unless otherwise noted.

### 2.7. Exosomes Isolation and Western Blotting

B16F10 cells, purchased from ATCC, were cultured overnight in DMEM high glucose (Gibco) + 10% FBS. Cells were then washed 3 times with 1x PBS and fresh serum-free DMEM was added and the cells were incubated further for 24 hours. The serum-free medium was collected and exosomes and microvesicles were isolated through a series of ultracentrifugation steps. In brief, the collected media were subjected to the following centrifugation steps, 600 ×g for 20 minutes and 10,000 ×g for 30 minutes, wherein the pellet was discarded and the supernatant was collected. Finally the supernatant was subjected to an ultracentrifugation step of 150,000 ×g for 3 hours. After ultracentrifugation, the pellet formed was washed in PBS. After the final washing, the pellet was resuspended in PBS and used for further analysis. Similarly, exosomes were also isolated from mouse breast cancer cell line 4T1 and human glioma cell line U87.

After harvesting the media, the cells were lysed in RIPA buffer containing protease and phosphatase inhibitors. The total protein content of cell lysates and exosomes was determined using the BCA protein assay kit from Thermo Scientific. Equivalent quantities of cell lysates, microvesicles, and exosomes were resolved onto SDS-PAGE gels and transferred to a PVDF membrane. The PVDF blot was blocked with 5% bovine serum albumin (BSA) in tris-buffered saline (TBS) containing 0.1% tween 20. The blots were then probed with the primary polyclonal antibodies against CD63 (SBI Biosciences, Palo Alto, CA) or beta-actin (Abcam, Cambridge, MA). The blots were then probed with horse radish peroxide (HRP) conjugated secondary antibodies (Santa-Cruz Biotechnology, Dallas, TX) against the primary antibodies. The blots were then developed using a chemiluminescence reagent and blots detected using the BioRad Chemidoc MP system (Hercules, CA).

### 2.8. Statistical Analysis

Mice were subjected to* in vivo* monitoring from 30 minutes up to 3 hours depending on the experimental task. To equalize the data changes due to vessel size, position of day-to-day tests, and mouse individuality, similar vessels in the mouse ear with diameters of 40–60 *µ*m were used for monitoring a minimum of two times at different laser positions with similar vessel size. Results are expressed as means plus/minus the standard error of at least three independent experiments (*P* < 0.05).* MATLAB 7.0.1* (MathWorks) was used for the statistical calculations.

## 3. Results and Discussion

### 3.1. Detection of Mimic CTPs* In Vitro* in a Capillary Tube and* In Vivo* in Control Mice

**Figure 2:**
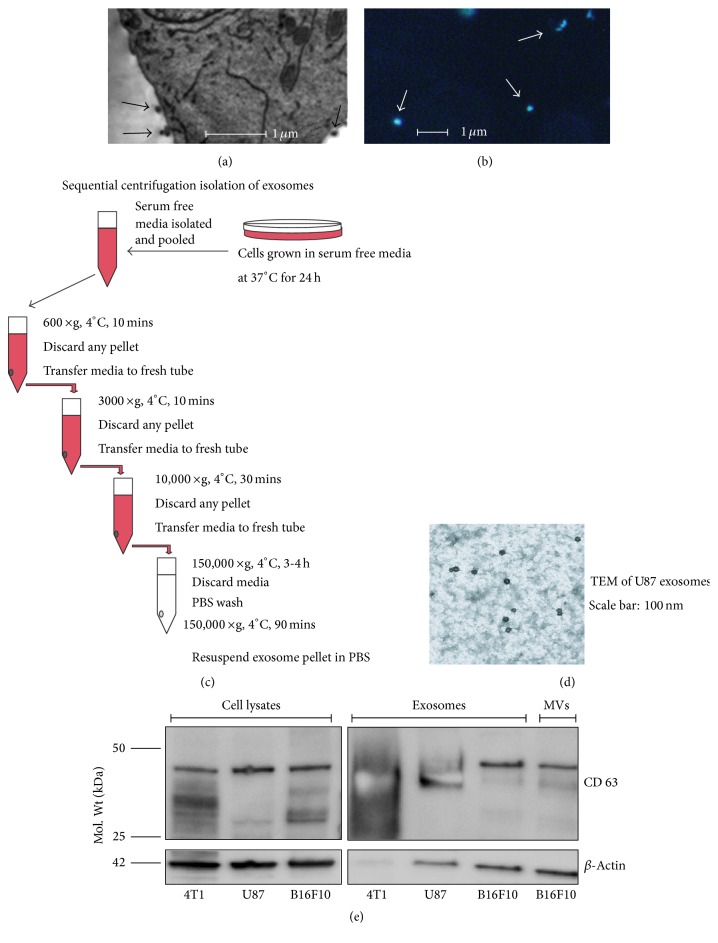
*In vitro* characterization and imaging of exosomes. (a) TEM image of B16F10 melanoma cell fragment with exosomes (arrows). (b) Dark field image of exosomes from B16F10 melanoma cells (light scattering contrast). (c) Schematic of exosome isolation. (d) TEM image of U87 exosomes. (e) Western blot of U87, 4T1, and B16F10 cell lysates and exosomes.

**Figure 3:**
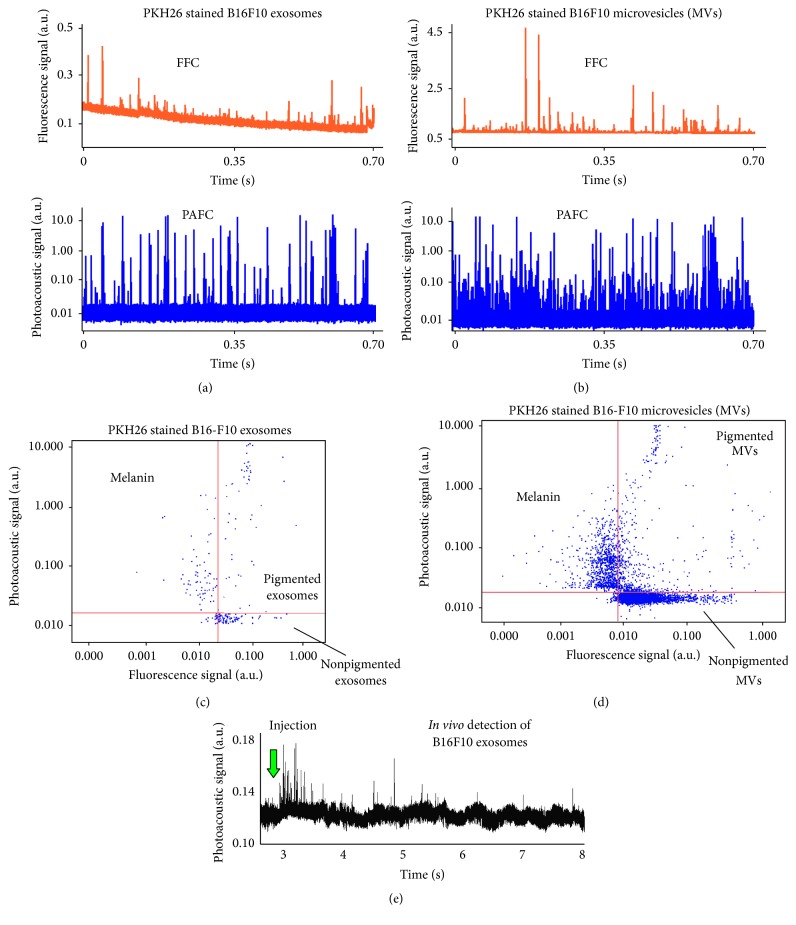
PA and fluorescence detection of CTPs in flow* in vitro* and* in vivo*.* In vitro* fluorescence PA signal traces of PKH26 dye stained exosomes (a) and microvesicles (b) from B16F10 melanoma cells in a 50 *µ*m capillary tube. 2D analysis of exosome (c) and microvesicle (d) detection in (a) and (b). (e)* In vivo* PA detection of exosomes from B16F10 cells which were intravenously (i.v.) injected in a healthy control mouse. PAFC laser parameters: 10 kHz pulse rate, 8 ns pulse width, and 30–50 mJ/cm^2^ energy fluence.

Melanin in melanoma cells has been used previously by our laboratory as an endogenously expressed PA contrast agent for the detection of CTCs* in vitro* and* in vivo* in both animal models and cancer patients [34, 35]. Based on this experience, we isolated exosomes and microvesicles from melanoma cells (B16F10) (Figures 2(a) and 2(b)) using well-established isolation procedures (Figure 2(c)) to verify the capability of the integrated PAFFC system to detect CTPs* in vitro*. TEM (Figures 2(a) and 2(d)) and dark field (Figure 2(b)) imaging revealed exosomes with sizes in the range of 60–90 nm. Some portions of exosomes were relatively dark suggesting the presence of melanin. We also observed rare, larger exosome clusters with sizes up to ~1 *µ*m. Further characterization of isolated exosomes and microvesicles was carried out using Western blot by probing for the exosomal marker protein CD63. CD63, a tetraspanin molecule, is highly enriched in exosomes in contrast to total cell lysate. The Western blot shows that, compared to the cell lysates, CD63 was present in abundant quantities (Figure 2(e)) in the isolated exosomes and also in microvesicles. Beta-actin was used as a loading control to depict relative quantities of protein samples loaded onto SDS-PAGE gels. Exosomes and microvesicles isolated from B16F10 cells were stained with PKH26 fluorescent dye (lipid membrane stain) and analyzed using both PAFC and FFC modules* in vitro* in a 50 *µ*m capillary flow tube. B16F10 exosome and microvesicle staining with the bright membrane fluorescence dye (PKH26) produced many fluorescence peaks (Figures 3(a) and 3(b)) and coincident PA transient signals (Figures 3(a) and 3(b)) generated by 820 nm pulse laser in the spectral range with no dye absorption (dye is absorbed in visible range only). Like conventional flow cytometry, the fluorescent PA data was presented in 2D plots (Figures 3(c) and 3(d)). The analysis of this data allowed us to identify three types of CTPs released from B16F10 cells: (1) nonpigmented CTPs (only fluorescence signals), (2) pigmented CTPs (both fluorescence and melanin-related PA signals), and (3) bare melanin particles (see Figures 3(c) and 3(d)). On average, the total ratio of pigmented CTPs was ~10%. However, more than 30% of CTPs containing melanin were also enveloped into a lipid bilayer like most of the exosomes. Such envelope may dramatically increase the diffusion rate for exosomes allowing them to easily penetrate into blood circulation and providing a natural source of early biomarkers for cancer diagnosis and monitoring.

Thus, some of the CTPs secreted from melanoma cells can be detected with PAFC in label-free mode. Indeed, intravenous injection of exosomes isolated from melanoma cells in control mice followed by the monitoring of exosomes in the ear of a mouse with PAFFC led to the appearance of PA peaks (Figure 3(e)). The clearance rate (lifetime) of these externally introduced particles (mimic CTPs) was around 10–15 minutes; however some signals appeared even after one hour during the continuous PA monitoring procedure. The number of PA signals and their amplitudes* in vivo* in the presence of background from skin and blood were lower compared to almost ideal optical condition* in vitro* (Figures 3(a) and 3(b)). Obviously, the conditions associated with light attenuation in biological tissues and background noise limit the sensitivity of most optical methods* in vivo*. The advantage of the PAFFC platform and especially the PAFC module is the potential to further improve the performance* in vivo*, in particular, by nonlinear amplification of PA signals from CTPs by the use of a picosecond laser which is optimal for the effective generation of PA signals from small absorbing targets like CTPs (acoustic confinement [34]) and by the use of an optimized acoustic detection system.

### 3.2. PA Detection of Real CTPs* In Vivo* Using Tumor-Bearing Mouse Models

To test the capability of PAFC to target CTPs in preclinical condition, we used an orthotropic xenograft mouse model of melanoma and metastatic breast cancer. These models were characterized by the early production of blood CTCs with the high probability for the appearance of CTPs. Melanoma CTCs and CTPs were detected in label-free mode using endogenously expressed melanin as a PA contrast agent, while breast cancer CTCs with GFP were molecularly targeted by GNR conjugated with folic acid [37]. As described in previous papers [34, 37], molecular targeting was optimized initially* in vitro*. In particular, for the detection of labeled breast cancer cells, we used laser energies that were a little below the label-free detection thresholds for breast cancer cells in control samples. Labeling specificity was provided through the bioconjugation of GNR-820 with folic acid for targeting of the surface folate receptors, which are highly expressed in breast cancer cells but almost absent in normal blood cells. We explored the dependence of labeling efficiency on nanoparticle concentration. Breast cancer cells were labeled by GNR-820 folic acid at concentrations ranging from 500 to 10,000 nanoparticles per one cell. Surprisingly, 500 GNR conjugates per one cell were even enough for effective PA detection of 30–40% cells* in vitro*. At 2000–3000 GNRs per one cell, the cells provided detectable PA signals in blood backgrounds. Labeling* in vivo* was performed by intravenous (i.v.) injection of GNR solution (100 *µ*L,  ~10^12^ GNRs/mL) into mouse blood circulatory system. Immediately after injection we observed the appearance of PA signals from GNR alone, mostly from their aggregates. Then, the PA signals quickly disappeared after ~5 minutes due to rapid clearance of GNR aggregates and appeared again from labeled cells after 20–30 minutes [34].

**Figure 4:**
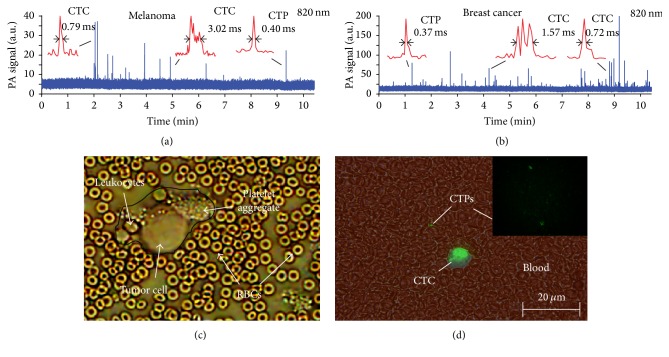
*In vivo* detection of CTCs and CTPs in tumor-bearing mouse models. (a) PA signal traces for melanoma animal model using B16F10 cells in label-free mode. Insets show typical peak shapes for CTCs and CTPs. (b) PA signal traces for breast cancer model using MDA-MB-231 cells labeled with conjugated gold nanoparticles. Insets show typical narrow peaks from CTPs and wider peaks from CTCs and/or CTP clusters. (c), (d) Images of CTPs in blood collected from melanoma (c) and breast carcinoma (d) tumor-bearing mice using cells with GFP.

We observed PA signals with narrow, medium, and wide widths associated with CTPs, CTC, and CTC aggregates, respectively (Figures 4(a) and 4(b)). Surprisingly, the amplitudes of PA signals from CTPs were only a little lower (3–5 times) than those from CTCs, which suggests a highly localized concentration of PA contrast agents in CTPs (e.g., melanin and GNRs). Image analysis of collected mouse blood confirmed* in vitro* the presence of CTCs and CTPs (Figures 4(c) and 4(d)).

The travel time of a CTC with a typical diameter (*d*_CTC_) of 12–25 *µ*m through the 3–5 *µ*m wide one laser beam spot (*d*_*B*_) determines the duration of a signal (Δ*t*) through the simple relation: Δ*t* ≈ (*d*_CTC_ + *d*_*B*_)/*V*_*F*_ (3–6 ms), where *V*_*F*_ is the flow velocity (3–7 mm/s in a mouse ear vessel). Thus, CTC (and CTP) size can be found if *d*_*B*_ and *V*_*F*_ are known [34, 38, 40]. Usually, *V*_*F*_ is not precisely known. Thus, in Figures 4(a) and 4(b), we can definitely operate with the widths of CTPs and CTCs and with some precaution of their size estimation using a single laser beam.

### 3.3. Analysis of CTC and CTC Sizes Using* In Vivo* Two-Beam Time-of-Flight Flow Cytometry

**Figure 5:**
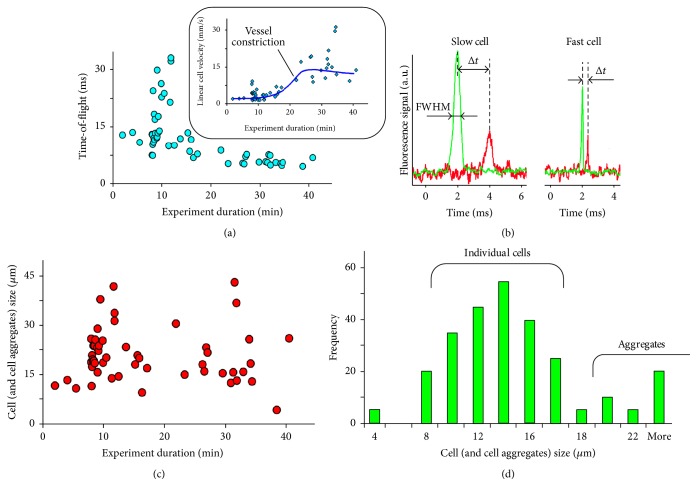
CTC size analysis using two-color two-beam* in vivo* PAFFC platform. (a) Time-of-flight data for cells after injection in a slightly constricted artery. Inset shows linear cells velocity measured from time delay (Δ*t*) between signals. (b) Typical fluorescence signals from slow (left) and fast (right) cells. Arrows indicate FWHM and Δ*t* delay parameters acquired for each cell. (c) Cell (and cell aggregates) size calculated from time-of-flight and cell velocity data. (d) Cell (and cell aggregates) size distribution histogram calculated from* in vivo* data.

 As mentioned in the previous subsection, time-of-flight data without the exact information on linear cell velocity cannot be used if there are some changes in a biological system over an extended period of time. This issue was explored using MTLn3 adenocarcinoma cell line expressing Dendra2 fluorescein protein and the FFC module of PAFFC.  Figure 5(a) shows how difficult it is to estimate the absolute cell size even for a uniform cell population with much less of a probability to identify small CTPs. Increase in blood flow velocity (Figure 5(a), inset) decreases the time-of-flight and introduces an error into an estimated cell size. To overcome this problem, we explored a two-beam schematic (Figure 1(c)) with which we can measure the delay between the two signal peaks (Δ*T*) created when a CTC or CTP sequentially crosses the two beams (Figure 1(d)). This enables us to calculate cell (object) velocity (*V*_*O*_ = *L*/Δ*T*) and thus increases the accuracy of cell size measurements (e.g., without the use of calibrated cell phantoms). For a cell producing signals from both channels (Figure 5(b)), as mentioned above, cell velocity can be calculated using only the delay between signals (experimental data) and the distance between the laser beams, *L* (constant hardware parameter). Indeed, our data indicated that in the same vessels there could be slow cells, having large time-of-flight signal duration (Δ*t* as FWHM of the peak) and a long interval between red and green peaks in the data recording, so that fast cells with narrow peaks are close to each other. Thus, from the cell (object) velocity, *V*_*O*_, and time-of-flight data, FWHM, one can calculate cell (object) size as *D*_cell_ = 2*V*_cell_FWHM; here a factor of 2 is used to calculate cell diameter.

In this case cell size data does not depend on flow velocity (Figure 5(c)). We performed analysis of cell size distribution by injecting fluorescent cells into blood circulation and monitoring transient signals using FFC. The acquired cells histogram data shows the presence of individual cells in flow and of few aggregates (Figure 5(d)). The average cell size measured* in vivo* (14 ± 1.4 *µ*m) using only a-priori acquired data on beams separation and laser beam width correlated well with the* in vitro* data on cell size for the same batch of cell, 12.8 ± 0.26 *µ*m, acquired using transmission microscopy on a slide. Our data indicates that some signals may be attributed to fluorescent microparticles or cell fragments having a size of just a few micrometers.

### 3.4. Monitoring of CTPs at Early Cancer Stages

**Figure 6:**
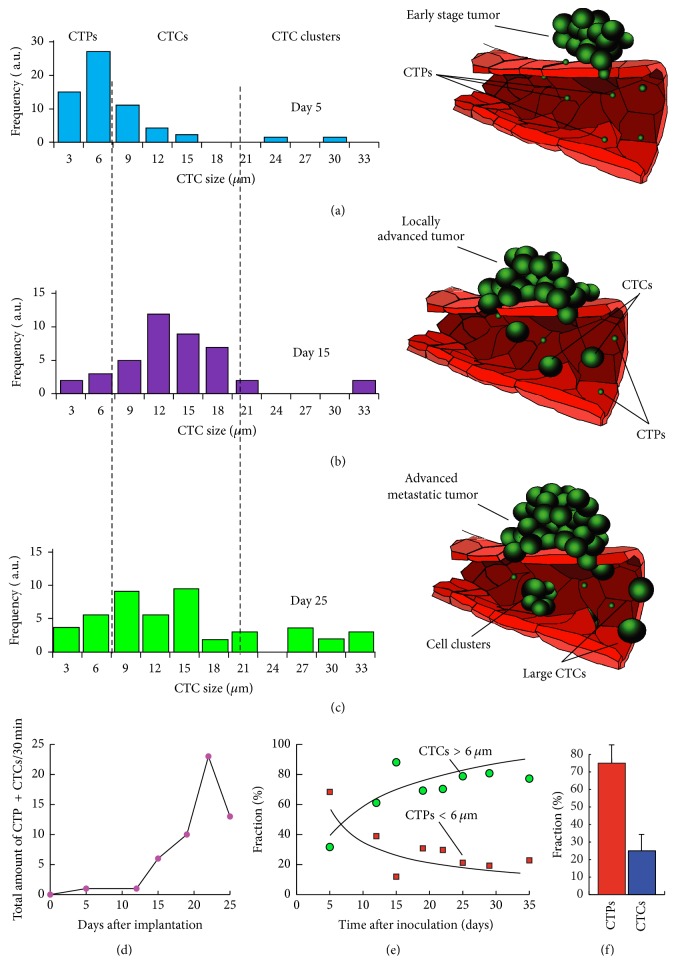
Monitoring of CTPs at early cancer stages. (a), (b), and (c) show histogram analysis of size frequencies during MTLn3 tumor development showing the prevalence of CTPs at day 5 and an increase in relative number of CTCs and clusters for days 15 and 25 (left) and the hypothesis and schematic of an early stage appearance of CTPs over CTCs in blood circulation (right). (d) Typical number of CTP/CTCs in circulation during tumor progression. (e) Proportion of CTPs (squares) and CTCs (circles) over time after tumor inoculation. (f) Proportion of CTPs and CTCs in carcinoma-bearing mouse at 3 days after tumor inoculation.

In order to develop an early cancer diagnosis tool based on the quantification of CTPs, it is important to confirm the original hypothesis that small tumor-associated particles appear earlier than CTCs in circulation and in much higher numbers due to a higher diffusion rate through healthy vasculature (early disease stage) than full-sized tumor cells. Two-color PAFFC operating in time-of-flight mode provides a unique opportunity to not only quantify CTP and CTCs in blood circulation, but also assess the size of circulating particles (Figures 6(a), 6(b), and 6(c) right). A mouse model featuring Dendra2-MTLn3 cells implanted into the mouse's ear was used in this study. These cells were expressing switchable Dendra2 protein and provided two-color fluorescence in the green and orange channels of the PAFFC system. The two-color time-of-flight PAFFC mode was used for both detection and size analysis of the circulating cells and cell fragments. Figures 6(a), 6(b), and 6(c) (left) demonstrate that the number of CTPs was substantially higher for the early stage of cancer development (day 5). At day 15 the relative number of signals from cells (9–18 *µ*m) was higher than of CTPs, and by day 25 a number of cell clusters started to appear in circulation. It should be noted that the actual number of CTPs can be much higher than the numbers detected here, as smaller cells and cell fragments have much lower fluorescence and it is much more difficult to detect such particles.* Ex vivo* analysis (similar to data in Figures 4(c) and 4(d)) revealed many exosomes with sizes in the range of 100–300 nm.

## 4. Conclusions

Detection and identification of CTPs, especially at an early disease stage, are still challenging because of their small size and heterogeneity, as well as the limited sensitivity and specificity of analytical methods. Our first-of-their-kind results demonstrate the high potential for* in vivo* multicolor PAFFC platform that integrates PA and fluorescence methods to detect CTPs. To verify the detection of CTPs, we have used the coincidence of PA and fluorescence signals (Figures 3(a) and 3(b)) using CTPs expressing melanin and labeled with PKH26 fluorescent membrane dye as PA and fluorescence reference signals. This integrated PAFFC (PAFC + FFC) technique can be considered a unique research tool that uses animal models to provide insight into the role of CTPs in cancer staging and prognosis, while PAFC has the potential for future clinical applications, especially in label-free noninvasive mode for the detection of melanoma. The focus of this paper is on the detection of CTPs* in vitro* and* in vivo* in animal models using endogenously expressed melanin, dyes, and genetically modified cells with fluorescent proteins as PAFFC contrast agents. Certainly, fluorescent proteins may enhance the preclinical research of CTPs; however, the use of such proteins is difficult in patients, if not impossible. In future experiments, we hope to use molecular targeting such as tumor specific antibodies and engineered nanoparticles to distinguish tumor-associated exosomes from host exosomes. Targeting CTPs using engineered nanoparticles is a good alternative but requires the reliable elimination of nonspecific interactions and has toxicity concerns.

In our current studies in tumor-bearing mouse models, we were able to distinguish CTPs with narrower peak widths and smaller amplitudes and single CTC or CTC aggregates with complex shapes and larger peak widths using advanced PAFFC diagnostic platform (Figures 4(a)–4(c)). Using PA detection of circulating B16F10 mouse melanoma cells in label-free mode and detection of labeled MDA-MB-231 breast cancer cells, we observed a dramatic difference in time it takes cells to cross the irradiated blood volume. The typical transition time for CTCs was 2–4 ms, while some signals appeared for only a short time of 0.1–0.5 ms. This indicates that small sized CTPs are present in flow. We also discovered that CTPs appeared at early stages of cancer progression and in larger numbers than CTCs (Figures 6(e)-6(f)). While the hypothesis that CTPs are shed from a primary tumor before CTCs has not been shown in the clinic yet, our findings support this link between CTPs and CTCs. Based on the obtained data, we suggest that CTPs can be valuable tools in cancer staging, prognosis, and determination of aggressiveness of the disease.

As mentioned, we cannot exclude aggregation (clumping) of CTCs and CTPs as well as the interaction of CTCs and potentially of CTPs with platelets. In particular, we have already observed CTC aggregates providing positive PA signals with some peaks having a broader width compared to single CTCs (e.g., [34] and Figures 4(a) and 4(b) in the current manuscript). We have also developed negative contrast PAFC agents, in which the platelet clumps or so-called white clots consisting of low absorbing platelets, fibrin, or white blood cells produce negative PA transient peaks in relatively strongly absorbing RBC background [34, 35]. Moreover, using this mode we observed CTC-platelet clumps providing a complex of positive-negative peaks [34]. Thus, our techniques can potentially monitor the clumping of pigmented CTP with platelets leading to positive-negative PA peaks.

The safety of PAFC diagnostic platform using relatively low laser energy fluence was verified in our previous publications and summarized in this review [34]. In our current study, we used similar laser parameters (e.g., 30–50 mJ/cm^2^) and thus we do not expect laser-induced CTC and CTP exosome photodamage. Nevertheless, the safety issue will be carefully examined in our future studies.

Label-free detection of CTPs is possible for at least one cancer type, melanoma, which could be an optimal model to explore the clinical potential of PAFC. We would like to emphasize that, for the first time, we have proposed a way to accurately measure the size of CTPs* in vivo* through the time-of-flight detection of CTPs between two laser beams (previously we and other laboratories estimated relative CTC sizes with the use of only one single beam) [38].

Hypothetically, our diagnostic platform can enhance the sensitivity of cancer diagnosis up to 10^6^-fold compared to that of existing* in vitro* CTC assays through the synergy of the increased volume of blood that can be examined with the* in vivo* PAFC platform (~10^3^ times) and the amplification of cancer markers in blood as a result of the potential for a single tumor cell to release many CTPs (~10^3^ times). The limitations of our first-generation* in vivo* PAFC platform include the low sensitivity for small targets and size controlling ≥0.5–1 *µ*m due to nonoptimized software which both did not allow us to achieve this dramatic potential improvement. On average, the percentage of detected pigmented exosomes was in the range of ~10%. Nevertheless, dramatically increased sensitivity may compensate for the missing or low pigmented cells. Moreover, more than 30% of CTPs that contained melanin also were enveloped into a lipid bilayer like most of the exosomes. Such envelope may dramatically increase the diffusion rate for CTPs which would allow them to easily penetrate into blood circulation and provide a natural source of early biomarkers for cancer diagnosis. We are confident that the sensitivity and specificity for detecting and identifying CTPs in blood can be significantly enhanced by the use of an advanced* in vivo* PAFFC-based platform that integrates high pulse repetition rate picosecond lasers, a two-beam time-of-flight schematic, and multicolor plasmonic probes.
